# Mammalian Expression and Mass Spectrometric Characterization of Highly Active Human Arylsulfatase A

**DOI:** 10.1002/biot.70312

**Published:** 2026-09-25

**Authors:** T. Pal, M. Dabarera, A. Dutta, G. Smolenski, B. D. Dickson, A. Williamson, W. Kelton

**Affiliations:** ^1^ Te Aka Mātuatua School of Science University of Waikato Hamilton New Zealand; ^2^ MS3 Solutions, Ruakura Research Centre Hamilton New Zealand; ^3^ School of Pharmacy and Biomedical Science University of Waikato Hamilton New Zealand

**Keywords:** Arylsulfatase A, formyl glycine modification, protein expression, sulfatases

## Abstract

The lysosomal enzyme Arylsulfatase A (ARSA) plays a critical role in hydrolyzing sulfatides from within cells. While gene therapies for ARSA replacement have recently been approved, challenges remain in recombinantly producing large amounts of active ARSA, especially for research purposes where generating stable cell lines is cumbersome. A major bottleneck is the need to convert the active‐site residue Cysteine at position 69 to formylglycine by a formylglycine‐generating enzyme (FGE). Further, transporter mannose‐6‐phosphate receptors (M6PR) are required to mediate active transport of ARSA out of the cell. To address these issues, we have optimized a three‐plasmid transient transfection approach that produces ARSA with active site conversion of over 70% as measured by mass spectrometry and is highly active in assays with a model substrate. We find the optimal ARSA:M6PR:FGE plasmid ratio to be 8:1:1, which balances active site conversion with total protein yield. This optimized protocol will assist in producing highly active ARSA for biochemical studies in research labs with access to standard cell culture facilities.

## Graphical Abstract and Lay Summary

A method for producing active human aryl sulfatase A by transient transfection of HEK293 cells. Accessory plasmids are included to enhance active site conversion and assist with expression/export.

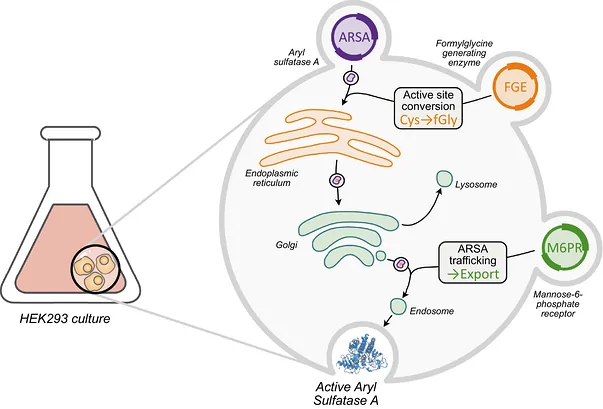

## 1 Introduction

Human Arylsulfatase A (ARSA) is an essential lysosomal hydrolase enzyme from the Arylsulfatase family and is responsible for the removal of sulfates from sulfatide. Functionally deleterious mutations in this enzyme cause severe pathogenic effects in vivo, with the deposition of sulfatide leading to progressive demyelination in metachromatic leukodystrophy (MLD) [1, 2], leading to severe dysphagia, regression of motor skills, seizures, hypotonia, ataxia, and peripheral neuropathy [3, 4]. Despite having an orphan disease status with an incidence of less than 1 in 40,000 births [5], there has nonetheless been long‐held interest in developing curative gene therapies for MLD. These efforts have culminated in the recent approval of the lentiviral‐based atidarsagene autotemcel (brand name Lenmeldy), which is currently listed as the most expensive drug in the world [6]. Beyond protein replacement therapies, there is growing interest in characterizing naturally occurring ARSA variants involved with MLD [7] and other neurodegenerative diseases like Parkinson's disease [8]. However, a longstanding challenge has been the recombinant production of active ARSA for research purposes. While bacterial systems can produce enzymes for sulfatase maturation (e.g., anaerobic sulfatase‐maturating enzymes [9]), eukaryotic systems preserve posttranslational modifications such as glycosylation, which have been shown to influence ARSA function [10, 11]. A number of methods with varying degrees of technical challenge have been developed to express Arylsulfatases in mammalian cells, including the use of retroviral transduction [12], cDNA transfection [13], and the generation of stably expressing cell lines [7, 14]. As a member of the Type I sulfatase family, active ARSA expression is further complicated by its dependence on an enzymatic post‐translational modification, where the active site cysteine residue, Cys‐69, is converted to a formylglycine (fGly; 2‐amino‐3‐oxopropionic acid) residue in the active state [15, 16, 17]. This fGly modification is required for the unique catalytic mechanism of sulfatases, where its hydrate oxygen acts as a nucleophile attacking the sulfur atom of the sulfate group, providing rate enhancements up to 10^26^‐fold (*k*_cat_/*k*_uncat_) [18]. Conversion to Cys‐69 fGly typically requires the co‐expression of Formylglycine‐generating enzyme (FGE) alongside ARSA unless endogenous intracellular FGE levels are relied upon during production. Quantifying the conversion of Cys‐69 to fGly is equally non‐trivial. The original discovery of the fGly modification relied on mass spectrometry [15] for detection, but conversion efficiencies are not generally reported in studies of ARSA activity assays against various substrates [19].

Previous ARSA expression studies have also demonstrated the requirement for mannose‐6‐phosphate receptors (M6PR) for efficient cell export. In normal cell function, intracellular trafficking of ARSA is dependent upon M6PR binding after the acquisition of mannose‐6‐phosphate post‐translational modifications in the Golgi apparatus [20]. Studies in baby hamster kidney cells showed a complete lack of soluble expression of ARSA unless co‐transfection of the 46 kDa human M6PR was included [11]. Here, we present a straightforward method for producing highly active ARSA by transient transfection of HEK293 cells. Correctly modified ARSA production and secretion are maximized by the co‐transfection of plasmids encoding both the 46 kDa M6PR and the FGE alongside ARSA. We further show that the active‐site conversion can be measured using mass spectrometry to quantify the fractional conversion of Cys‐69 to fGly‐69. These approaches will assist with standardizing ARSA activity assays across a broad range of substrates.

## 2 Methods

### 2.1 Gene Construct Design

All gene constructs were ordered from Twist Biosciences (USA) in pTwist CMV BetaGlobin WPRE Neo vectors with NotI/ClaI insertion sites. All gene inserts are described in Table S1. For ARSA, we chose transcript variant 1 as a human reference sequence and selected residues 1–507 (NCBI accession number: NM_000487.6). FGE enzyme residues 1–374 were taken from transcript variant 1 (NCBI accession number: NM_182760.4). Finally, the M6PR residues 1–277 were taken from transcript variant 1 (NCBI accession number: NM_002355.4). All sequences were used in their native form without codon optimization. Each plasmid was cloned into chemically competent DH5α *E. coli* cells, grown overnight at 37°C in LB broth containing 100 µg/mL carbenicillin, and extracted using the Endo‐free Plasmid DNA mini kit protocol (Omega Bio‐Tek).

### 2.2 Protein Production

Expi293F HEK cells were cultured in Expi293 PRO Expression medium (ThermoFisher) and were expanded to 7 × 10^6^ viable cells/mL. Cells were transfected with Expi293 PRO Transfection Kit (ThermoFisher) at a density of 5 × 10^6^ cells/mL with 1 µg of plasmid per mL of culture according to the manufacturer's instructions. The transfected cells were incubated for 6 days at 37°C with 8% CO_2_ and 125 rpm shaking speed on an orbital platform (ThermoFisher 88881102). The cells were harvested by centrifugation of the culture medium at 6000 × g for 40 min at 4°C, and the supernatant was filtered through a 0.45 µm filter and then through a 0.22 µm filter. Proteins were purified using a Bio‐Rad NGC chromatography system and a 5 mL Strep‐TactinXT 4Flow column (IBA Biosciences) following the manufacturer's instructions. Briefly, the column was operated at a flow rate of 2 mL/min with binding buffer (100 mM Tris‐HCl, pH 8.0; 150 mM NaCl; 1 mM EDTA). Samples were loaded onto the column, followed by washing with 10 column volumes (CV) of binding buffer. Bound enzyme was eluted with a 0%–100% gradient of elution buffer over 4 CV (100 mM Tris‐HCl, pH 8.0; 150 mM NaCl; 1 mM EDTA; 50 mM biotin) followed by a 2 CV isocratic wash at 100% elution buffer. The column was regenerated by sequential washes with 3 CV of distilled water, 3 CV of 50 mM NaOH, and 3 CV of distilled water, followed by equilibration with 8 CV of binding buffer. Absorbance at 280 nm was used to determine protein concentrations on a Denovix Spectrophotometer with an $A_{280nm}^{0.1\%}$ coefficient of 0.925. Purified ARSA was concentrated using centrifugation with 10 kDa Amicon Ultra Centrifugal Filters (Merck Millipore). The filter units were then centrifuged at 4000 × g for 15 min at 4°C and buffer‐exchanged into phosphate‐buffered saline (PBS) at pH 7.36.

### 2.3 SDS‐PAGE and Western Blotting

SDS‐PAGE was performed using 12% polyacrylamide gels. Sample volumes of 15 µL were mixed with 5 µL of loading dye and heated at 95°C for 5 min before loading. As a reference, a lane containing 8 µL of Precision Plus Protein Unstained Protein Standard (Bio‐Rad) was also loaded into each gel. The gels were run at 120 V and stained with Coomassie blue for visualization. For Western blotting, unstained gels were transferred to a polyvinylidene difluoride (PVDF) membrane at 20 V overnight. The membrane was then blocked with 20 mL of PBS‐blocking buffer containing 2% *w/v* skim milk powder, incubated for 1 h at room temperature with gentle shaking, and washed three times for 5 min with PBST (PBS with 0.05% *w/v* Tween). Ten milliliters of Strep‐Tactin HRP (1:100, IBA Biosciences) was added to the membrane and incubated at room temperature for 1 h. After washing twice with PBST buffer for 1 min at room temperature, followed by two additional washes with PBS buffer for 1 min, visualization was performed by the addition of ECL Western Blotting substrate (ThermoFisher). Western blot band densities were quantified using ImageJ (Fiji) [21]. For each lane, the integrated density of the protein band was measured, and the background signal was subtracted using an area of the blot free of bands. Normalization was performed relative to a 5 µg control loading of previously purified ARSA protein. Data were assessed for normality using the Shapiro–Wilk test and Q–Q plots, and homogeneity of variance was checked using Levene's test. Statistical analysis was performed using one‐way ANOVA, followed by all pairwise comparisons with the two‐stage step‐up method of Benjamini, Krieger, and Yekutieli [22].

### 2.4 Mass Spectrometry Analysis

Peptide ionizing efficiencies were evaluated with chemically synthesized control peptides FTDFYVPVSLCTPSR and [fGly]FTDFYVPVSLTPSR (Genscript). Each peptide was reconstituted in 20% acetonitrile buffer at 1 mg/mL. To test the ionizing efficiency of the unmodified FTDFYVPVSLCTPSR with and without Cysteine acetylation, 10 µL of the unmodified peptide was added to 40 µL of 25 mM ammonium bicarbonate with 10 mM DTT at pH 7.8. Subsequently, 20 µL of iodoacetamide at 500 mM was added with another 30 µl of 25 mM ammonium bicarbonate. After 45 mins in the dark at 37°C, 90 µL of 25 mM ammonium bicarbonate and another 10 µl of 500 mM iodoacetamide were added for a further hour. This solution and non‐acetylated peptides were diluted to a final concentration of 1 ppm for injection. To analyze recombinant ARSA samples, protein bands from G‐250 Coomassie‐stained SDS‐PAGE gels were excised and incubated overnight with wash buffer (25 mM ammonium bicarbonate in 50% acetonitrile) to remove the stain. The gel pieces were then washed with fresh wash buffer and dehydrated using 100% acetonitrile. Next, each gel fragment was incubated with 10 mM DTT in 25 mM ammonium bicarbonate pH 7.8 for 30 min at 37°C, then with 55 mM iodoacetamide in 25 mM ammonium bicarbonate in the dark at room temperature for 60 min to reduce and alkylate the cysteine residues. The gel pieces were digested overnight at 37°C with 10 µL of 0.1 µg/µL trypsin in 25 mM ammonium bicarbonate containing 5% acetonitrile. Digested peptides were extracted from the gel pieces by sonication for 10 min in 0.2% formic acid/40% acetonitrile. Sonication was repeated a second time with fresh extraction buffer, and a final wash with 100% acetonitrile was performed to remove all remaining liquid from the gel. The liquid from all extraction steps was combined into a single tube for lyophilization. The dried peptide samples were reconstituted in 50 µL of 0.2% formic acid in 5% acetonitrile and analyzed by LC‐MS/MS using an Orbitrap Exploris 480 Hybrid mass spectrometer (Thermo Scientific). Peptide and protein identification was performed using PEAKS Studio v12.5 against the UniProt human proteome database (UP000005640). The spectra were also searched for alternative forms of fGly, including reduced, oxidized, and hydrated species. The relative abundance of fGly was determined from extracted ion chromatogram peak areas corresponding to the unmodified peptide FTDFYVPVSLCTPSR (*m/z* 866.4273), the IAA‐modified peptide FTDFYVPVSLC(+57.02)TPSR (*m/z* 894.9377), the fGly‐containing peptide FTDFYVPVSLC(−17.99)TPSR (*m/z* 857.4299), and its hydrated form FTDFYVPVSLC(+0.0175)TPSR. The proportion of fGly‐modified peptide was calculated as the sum of the fGly‐containing and hydrated fGly species relative to all detected peptide species, with a correction factor applied to the fGly‐ and IAA‐modified peptides to account for observed differences in ionization efficiencies of the synthetic peptide controls. This correction factor was calculated by taking the ratio of the area under the curve for the fGly‐ and IAA control standards. Briefly, raw data were analyzed using Freestyle software v1.6.75.20 (Thermo Scientific) to extract and visualize the specific peptide species. The Genesis peak detection algorithm was used to integrate peaks and obtain relative areas under the curve for each peptide species.

For the percent fGly modification, a normality test was performed (*n* = 3) using the Shapiro–Wilk test and Q–Q plots, and homogeneity of variance was checked using Levene's test. Statistical analysis was performed using one‐way ANOVA followed by all pairwise comparisons with the two‐stage step‐up method of Benjamini, Krieger, and Yekutieli.

### 2.5 Arylsulfatase Assays

Purified ARSA (0.2 and 0.1 µg) was incubated with 8 µL of 6.25 mM p‐nitrocatechol Sulfate (p‐NCS, Merck, catalogue no. N7251) and 40 µL of 200 mM sodium acetate buffer (pH 5.0) at 37°C over timescales of 0, 1, 5, 10, and 15 min in a total reaction volume of 50 µL. The enzymatic reaction was quenched by adding 100 µL of 1.0 M NaOH to each well. Finally, the absorbance was measured using a SpectraMax M4 (Molecular Devices) at 515 nm. The commercially available sulfatase from Helix pomatia (Sigma, S9626) was included as an assay control to verify functionality and was not used for direct comparison with the activity of our recombinant ARSA. All three ARSA production conditions were normalized to 1 µg of ARSA protein mass, and we calculated the specific activity in units of mU/µg as [µmol] p‐nitrocatechol per [µg] ARSA per [min]. Each assay was performed in triplicate. Blanks were measured by using PBS instead of the sample. The sample background was determined by adding 100 µL of 1.0 M NaOH before the absorbance was measured, which was used to subtract from the measured enzyme activities of each sample. The slope of a scatter plot with a linear regression was used to assess the specific activity over the time course.

## 3 Results and Discussion

**FIGURE 1:**
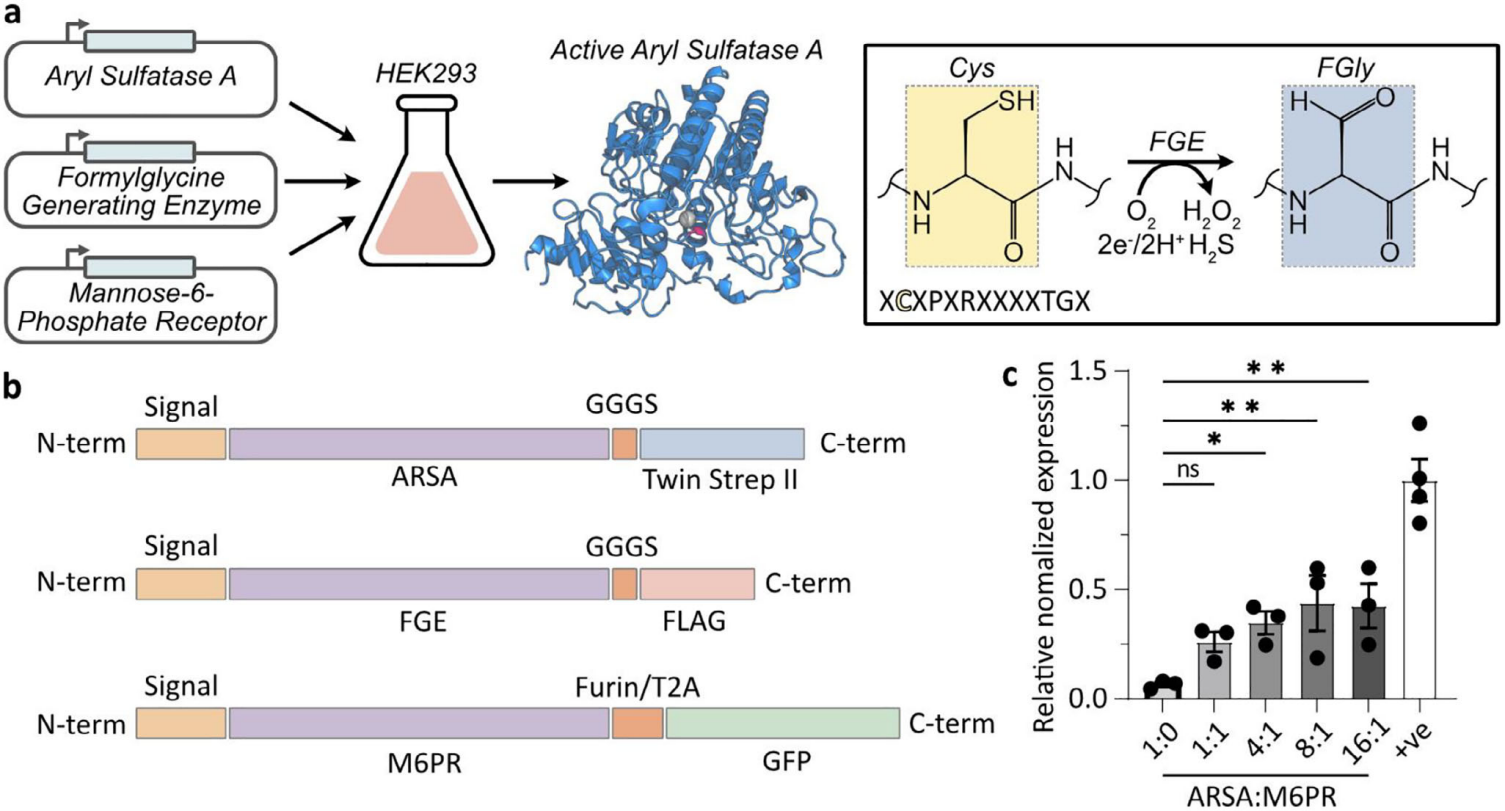
Optimization of ARSA expression yields. (a) Transient expression workflow of co‐transfection using ARSA, FGE, and M6PR (ARSA crystal structure PDB: 1AUK [23]). FGly modification mediated by FGE at Cys‐69 is required for enzyme activity [24]. (b) Design of ARSA, FGE, and M6PR expression constructs. (c) ARSA supernatant expression level analysis by Western blot using Strep‐Tactin HRP. Densitometry analysis was used to normalize band intensities to 5 µg of a previously purified in‐house batch of ARSA (+ve). Data are presented as the mean ± SEM (*n* = 3). A one‐way ANOVA was performed before pairwise comparisons between all samples using the Benjamini, Krieger, and Yekutieli two‐stage step‐up method (^***^ *p* < 0.001, ^**^ *p* < 0.01, ^*^ *p* < 0.05).

A series of three expression plasmids were designed to express the human ARSA, FGE, and 46 kDa M6PR proteins (Figure 1a). The ARSA sequence used the native N‐terminal signal peptide but was modified at the C‐terminus with a 28‐amino‐acid twin‐strep tag including an internal Gly‐Gly‐Gly‐Ser linker. Likewise, the FGE enzyme was altered to include a C‐terminal FLAG tag, and the M6PR was fused to a C‐terminal furin/T2A skipping peptide followed by GFP for detection of transfection (Figure 1b). Initial transient transfection tests in HEK293 cells with the ARSA plasmid alone produced very low levels of recombinant expression. To improve protein yields, we first optimized secreted ARSA yields by co‐transfecting with various ratios of the M6PR‐encoding plasmid (1:1 ARSA:M6PR to 16:1 ARSA:M6PR). This plasmid contains a GFP reporter cassette driven by a T2A skipping peptide, enabling easy confirmation of transfection via fluorescence at low ratios relative to the ARSA plasmid. We found that including even a small fraction of co‐transfected M6PR improved expression levels of soluble ARSA (One‐way ANOVA F(DFn, DFd), F(5, 13) = 15.49; *p* <.0001) detected in the cell culture supernatant by Western blot (Figures 1c and S1; Table S2). We speculate that M6PR facilitates endosomal trafficking of ARSA and promotes its extracellular routing, consistent with the mechanism proposed by Chao et al. [11]. As a result of these findings, a conservative midpoint ratio of 8:1 of ARSA:M6PR transfected plasmids was selected for all future experiments.

To mediate efficient fGly modification of the Cys‐69 residue in the ARSA active site, we optimized the ratios of co‐transfected FGE plasmid (ARSA:M6PR:FGE).

ARSA protein was chromatographically purified from each expression experiment, run on SDS‐PAGE, and the resulting trypsin‐fragmented peptides were analyzed by mass spectrometry. We found that including the FGE enzyme in co‐transfections reduced protein yields, and this effect was more pronounced at higher FGE‐to‐ARSA ratios. In our final expression trial, we obtained protein yields of 14.49 µg/mL of transfection culture for 8:1:0 ARSA:M6PR:FGE co‐transfections, 16.64 µg/mL for 8:1:1 ARSA:M6PR:FGE, and 11.4 µg/mL for 8:1:2 ARSA:M6PR:FGE after all purification steps. All transfections were undertaken at a 30 mL scale, but we note that transient transfection systems can readily be scaled to produce the desired amount of protein. Prior expression approaches have reported yields of ∼100 µg per 2 L batch using retroviral approaches [12] (Table 1).

**TABLE 1:** Previously reported methods for recombinant ARSA expression.

Expression system	Procedure	Yield	Reported activity	Reference
C2C12 cells	Retroviral transduction	100 µg/L	1.52 mU/µg (MUS substrate)	Martino et al., 2005 [12]
CHO‐k1 cells	Chemical‐based pcDNA3 plasmid transfection	Absolute yield not reported	45 mU/µg	Simonis et al., 2019 [25]
HEK293T	Chemical‐based pAAV9 plasmid transfection	Absolute yield not reported	∼18 nmol/h/mg	Mullagulova et al., 2023 [26]

**FIGURE 2:**
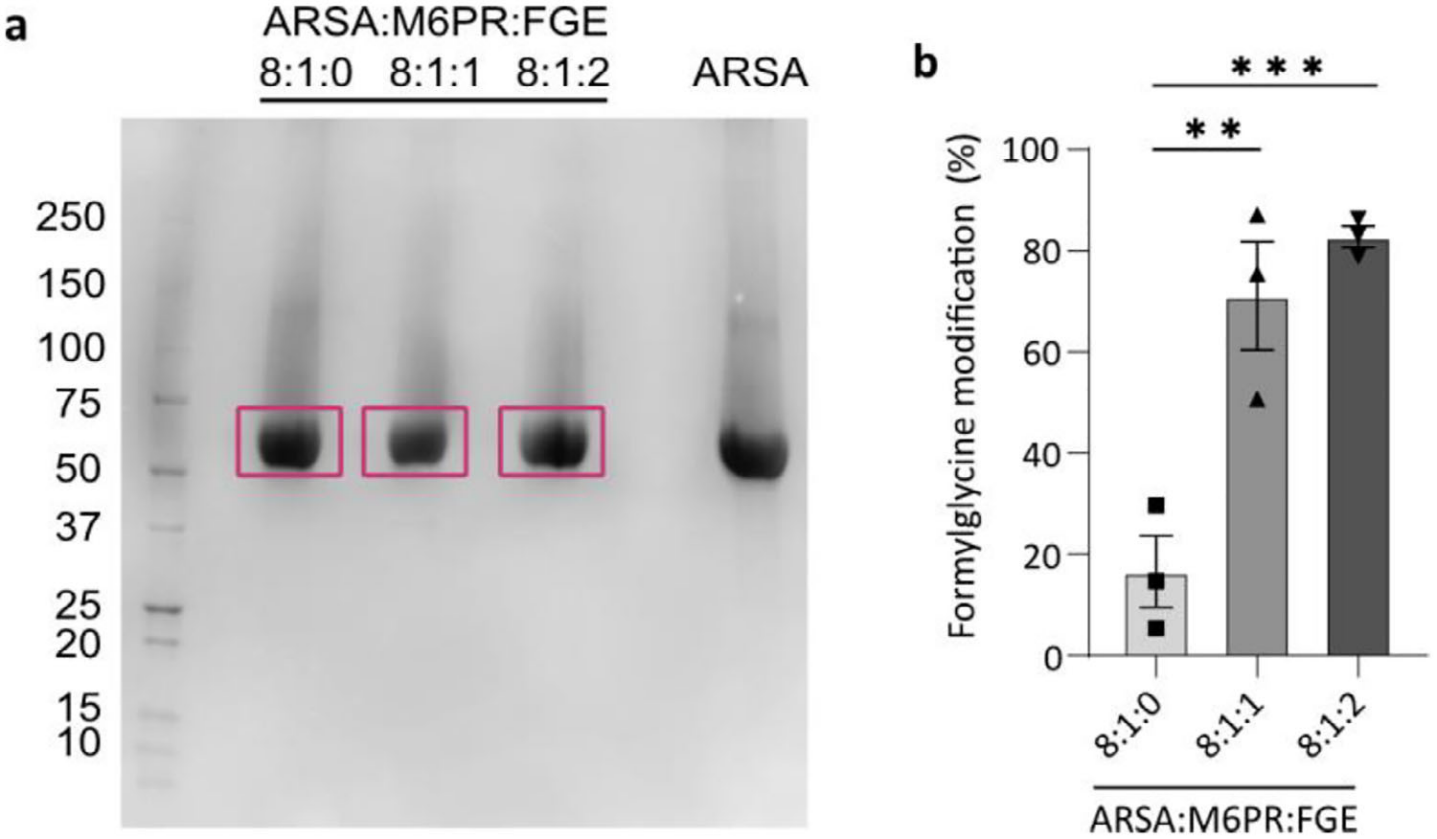
Optimisation of ARSA active site Cys‐69 conversion. (a) 12% SDS‐PAGE analysis of ARSA expression at various ratios of ARSA:M6PR:FGE plasmids as compared to a 5 µg ARSA loading control. Representative gel is shown from replicate data (*n* = 3). Bands highlighted in boxes were taken for mass spectrometry analysis. (b) Percentage conversion of the ARSA active site Cys‐69 to fGly by mass spectrometry analysis. ARSA was digested with trypsin prior to analysis. Data are presented as the mean ± SEM (*n* = 3), and a one‐way ANOVA was performed prior to pairwise comparisons between all samples using the Benjamini, Krieger, and Yekutieli two‐stage step‐up method (^***^ *p* < 0.001, ^**^ *p* < 0.01, ^*^ *p* < 0.05).

Although human ARSA has a predicted molecular mass of about 53 kDa, we observed the protein runs at an apparent molecular weight of 62 kDa on SDS‐PAGE, consistent with other work and indicative of posttranslational glycan modification [12] (Figure 2a). We used mass spectrometry to quantify the fractional conversion of the active site Cys‐69 to fGly. First, ionization efficiencies of diagnostic peptides were evaluated using synthetic Cys‐ and fGly‐containing peptides (Figure S2). We observed that acetylation was required for high‐efficiency ionization of peptides containing Cys‐69 at the enzyme active site (Table S3). Next, we ran recombinant ARSA samples isolated from SDS‐PAGE gel slices and observed a higher‐than‐expected fraction of unacetylated peptides, suggesting that modified and unmodified peptides exhibit markedly different analytical responses in complex digest matrices. While we have not been able to definitively confirm the cause of this discrepancy, background peptide effects and matrix‐dependent biases are well documented in proteomic LC‐MS analyses [27]. We further searched for modified forms of fGly and identified a small proportion of the hydrated species, which is commonly observed for aldehydes in aqueous solutions. After correcting for differences in the ionization efficiencies of the acetylated Cys and fGly modified peptides, and accounting for fGly hydration, recombinant ARSA showed a substantial increase in Cys‐69 modification from a baseline of 16.6% ± 10.0% without FGE plasmid transfection to 82.8% ± 3.0% using an ARSA:M6PR:FGE plasmid ratio of 8:1:2 (Figures 2b and S3; Table S4) (one way ANOVA F(DFn, DFd) = F (2, 6) = 22.05; *p* = 0.002; Table S5). A key limitation of our approach is a small replicate number leading to a relatively high standard error of the mean, particularly for the 8:1:0 ARSA:M6PR:FGE baseline. Nonetheless, these percentage conversions are higher than comparable production methods where FGE is not included during transfection [25]. We also observed unexpected but repeatable modification of surface‐exposed Cys‐294 (motif: CSGLL) away from the catalytic site (Figures S4–S6), although this modification does not appear to have a detrimental effect on the enzyme activity.

Collectively, our findings suggest a trade‐off between expression level and active‐site conversion. Prior studies have quantified the conversion of Cys‐69 in the active site at around 20% for production by in vitro translation [16] and between 60%–90% when made recombinantly [15]. It remains unclear what fraction of Cys is converted in endogenous ARSA produced by human cells in vivo, but for the purposes of in vitro work, a high fGly conversion rate is important, as ARSA enzymatic activity progressively declines post‐expression. We observed a progressive loss of ARSA activity in samples stored at 4°C (data not shown) and therefore recommend snap freezing in liquid nitrogen for long‐term storage.

**FIGURE 3:**
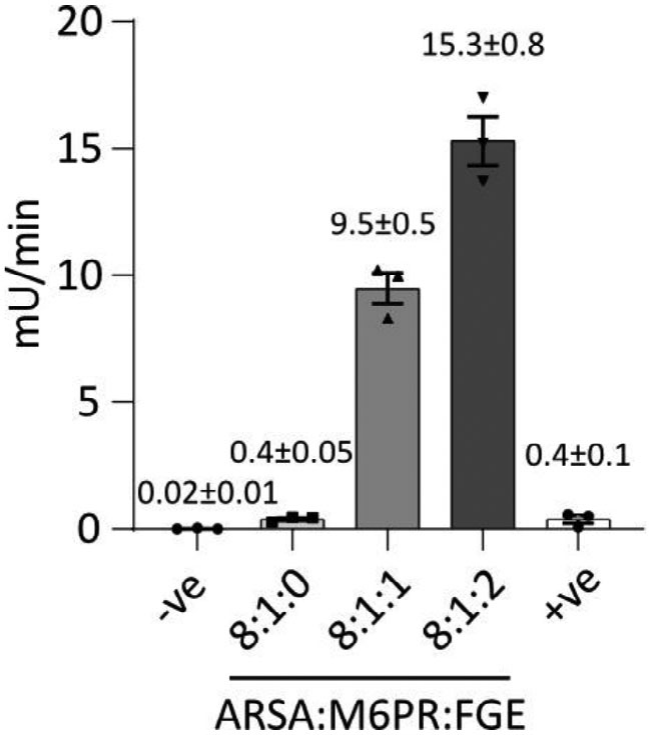
Specific activity of recombinant ARSA enzyme using p‐nitrocatechol sulfate as a substrate. The specific activity was calculated from the initial reaction rate obtained by linear regression of product formation over time in assays performed with 0.2 µg enzyme. Bars represent the mean specific activity, while Individual points are technical replicates (*n* = 3). Error bars indicate the standard error of the mean. Commercially available sulfatase from Helix pomatia was used as a positive control with each assay.

Finally, we confirmed the activity of ARSA co‐expressed with FGE (ARSA:M6PR:FGE ratios of 8:1:1 and 8:1:2) as compared to ARSA without high levels of fGly modification (ARSA:M6PR:FGE ratio of 8:1:0) by measuring the conversion of p‐nitrocatechol sulfate to p‐nitrocatechol (Figure 3). The heterologous co‐expression of FGE increased the ARSA specific activity relative to the natively modified control (Figures 3 and S7; Table S6). A difference in specific activity correlates with a higher proportion of modified fGly (Figure 2b). Conditions with high fGly modification at Cys‐69 (i.e., groups 8:1:1 and 8:1:2) showed an activity of 9.5 mU/µg and 15.3 mU/µg, respectively, while ARSA with low fGly modification at Cys‐69 (i.e., the 8:1:0 group) demonstrated an activity of 0.39 mU/µg, approximately 40 times lower than that of the enzymes with high fGly modification. Based on this enzyme assay data, we recommend transfecting with ARSA:M6PR:FGE plasmids at an 8:1:1 ratio to strike a balance between fGly conversion and ARSA expression yield. We further compared the activity of our enzymes to previously reported recombinant expression approaches (Table 1). Despite significant differences in the methods used to assay activity (e.g., direct assaying of cell supernatant or use of a different substrate), the values we report have reasonable alignment with prior observations.

## 4 Conclusion

Reproducible expression of highly active ARSA in sufficient quantities for enzymatic characterization is challenging, in part due to the requirement for posttranslational modification of the crucial fGly‐69 catalytic residue. Here, we have optimized a three‐plasmid co‐transfection approach to reliably produce ARSA with active‐site conversion from Cys‐69 to fGly‐69, as confirmed by mass spectrometry and specific activity assays. This method simplifies existing production schemes by adopting transient transfection, which is accessible to most laboratories with standard cell culture facilities. Further improvements in ARSA yield may also be possible by foregoing the GFP reporter gene in the M6PR plasmid or by using higher‐yielding transient transfection systems as they become available. Given that other sulfatases, such as ARSB, share the conserved FGE recognition motif (CXPXR), future studies could explore whether this recombinant production strategy can be extended to other sulfatase enzymes. Ultimately, the production strategy described here is scalable and will contribute to the standardized production of ARSA for further in vivo studies.

## Author Contributions

**T. Pal and M. Dabarera**: contributed equally to this work. **B. D. Dickson, A. Williamson,** and **W. Kelton**: conceived of and designed the study. **T. Pal, M. Dabarera, A. Dutta, G. Smolenski, and W. Kelton**: performed laboratory experiments. **T. Pal, M. Dabarera, and W. Kelton**: wrote the manuscript. All authors provided feedback and revisions for the final version of the manuscript.

## Funding

This work was supported by a University of Waikato seed fund grant to B.D., W.K., and A.W.

## Conflicts of Interest

The authors declare no conflicts of interest.

## Supporting information




**Supporting File**: biot70312‐sup‐0001‐SuppMat.pdf.

## Data Availability

All data supporting the findings of this study are available within the article and its Supporting Information.
