# Supplementary material for: Mammalian Expression and Mass Spectrometric Characterization of Highly Active Human Arylsulfatase A
Source: Biotechnol J. 2026 Sep 25;21(9):e70312. doi: 10.1002/biot.70312 (PMC13613566; doi:10.1002/biot.70312)
Supplement: Supplementary file 1 [file BIOT-21-e70312-s001.pdf]

Mammalian expression and mass spectrometric characterisation of highly active Aryl Sulfatase A

T. Pal, M. Dabarera, Dutta. A, G. Smolenski, B. D. Dickson, A. Williamson, and W. Kelton

Supplementary Material

Supplementary Tables

Supplementary Table S1. Gene sequences used in this work. Bold highlights indicate start and stop codons, underlined sequences are NotI/ClaI restriction enzyme sites used for cloning into pTwist CMV BG WPRE Neo vectors. In light blue is the Twinstrep tag, orange is the GGGGS Flag tag, and in purple is a Furin – T2A – superfold GFP expression reporter.

>ARSA	GCGGCCGCCC**ATG**GGCGCCCCACGATCTCTTCTGCTTGCGCTTGCCGCCGGACTCGCTGTGGCTCGCCCACCTAATATAGTTCTTATTTTCGCAGATGATTTAGGGTACGGTGATCTTGGATGTTACGGACATCCTTCTTCAACAACCCCTAATTTGGATCAATTGGCTGCCGGCGGCCTCAGATTTACCGACTTTTATGTACCCGTTTCACTTTGTACTCCTTCACGGGCAGCACTTCTCACTGGGCGTCTGCCCGTAAGGATGGGGATGTATCCCGGGGTGTTGGTCCCATCCAGCCGAGGCGGACTCCCACTCGAAGAAGTTACAGTCGCAGAGGTACTCGCAGCTAGAGGATATCTGACTGGCATGGCGGGTAAATGGCATCTGGGAGTCGGCCCCGAAGGAGCGTTTCTTCCACCGCACCAAGGATTTCACAGGTTCCTGGGAATACCTTATTCACATGATCAAGGGCCGTGTCAAAATCTCACATGTTTTCCCCCAGCTACCCCATGTGATGGCGGATGCGATCAAGGGCTCGTGCCAATTCCCTTACTGGCTAATCTTTCAGTCGAAGCTCAACCACCGTGGCTCCCTGGGTTAGAAGCTCGTTATATGGCCTTTGCACACGATCTGATGGCGGATGCTCAAAGACAAGACAGGCCATTCTTTCTCTATTACGCATCACATCATACACATTATCCCCAATTTTCTGGCCAATCATTCGCTGAACGGTCTGGGAGAGGCCCTTTCGGCGATTCTCTCATGGAACTCGACGCTGCCGTCGGCACGCTCATGACTGCGATTGGCGATCTCGGACTCTTGGAAGAAACCTTAGTGATCTTTACCGCCGATAACGGGCCCGAAACAATGAGAATGAGTAGGGGCGGTTGTTCAGGCCTGCTGAGATGCGGCAAAGGCACCACATATGAAGGCGGAGTGCGGGAACCCGCGCTGGCTTTCTGGCCTGGGCACATAGCGCCTGGGGTCACACATGAATTAGCATCCAGTTTAGATTTACTCCCAACTTTGGCTGCACTCGCAGGAGCACCCTTACCAAACGTTACACTGGACGGGTTCGATCTTTCCCCTCTTTTACTTGGTACTGGAAAATCACCGCGACAAAGCTTGTTCTTTTATCCCTCTTATCCTGATGAAGTGCGGGGAGTGTTCGCAGTTAGAACAGGGAAATATAAAGCACATTTCTTTACTCAAGGGTCCGCACATAGCGACACAACGGCCGATCCAGCATGTCATGCAAGTTCTTCCCTCACAGCGCACGAACCGCCTTTGCTTTACGATCTTTCAAAAGATCCGGGAGAAAATTATAATTTACTCGGCGGAGTCGCAGGCGCTACGCCCGAAGTCCTTCAGGCTTTGAAGCAACTGCAATTGTTGAAAGCTCAACTGGATGCCGCCGTAACTTTTGGGCCATCTCAAGTTGCACGCGGTGAAGATCCAGCATTGCAAATATGTTGCCACCCCGGATGTACTCCAAGGCCTGCCTGTTGTCACTGTCCTGACCCTCACGCCTGGTCCCATCCTCAATTCGAAAAGGGCGGCGGCTCCGGCGGTGGATCTGGTGGGTCCGCTTGGTCACATCCTCAGTTCGAAAAA**TGATAG**ATCGAT
>FGE	GCGGCCGCC**ATG**GCTGCGCCCGCACTAGGGCTGGTGTGTGGACGTTGCCCTGAGCTGGGTCTCGTCCTCTTGCTGCTGCTGCTCTCGCTGCTGTGTGGAGCGGCAGGGAGCCAGGAGGCCGGGACCGGTGCGGGCGCGGGGTCCCTTGCGGGTTCTTGCGGCTGCGGCACGCCCCAGCGGCCTGGCGCCCATGGCAGTTCGGCAGCCGCTCACCGATACTCGCGGGAGGCTAACGCTCCGGGCCCCGTACCCGGAGAGCGGCAACTCGCGCACTCAAAGATGGTCCCCATCCCTGCTGGAGTATTTACAATGGGCACAGATGATCCTCAGATAAAGCAGGATGGGGAAGCACCTGCGAGGAGAGTTACTATTGATGCCTTTTACATGGATGCCTATGAAGTCAGTAATACTGAATTTGAGAAGTTTGTGAACTCAACTGGCTATTTGACAGAGGCTGAGAAGTTTGGCGACTCCTTTGTCTTTGAAGGCATGTTGAGTGAGCAAGTGAAGACCAATATTCAACAGGCAGTTGCAGCTGCTCCCTGGTGGTTACCT

	GTGAAAGGCGCTAACTGGAGACACCCAGAAGGGCCTGACTCTACTATTCTGCACA<br>GGCCGGATCATCCAGTTCTCCATGTGTCCTGGAATGATGCGGTTGCCTACTGCACT<br>TGGGCAGGGAAGCGGCTGCCCACGGAAGCTGAGTGGGAATACAGCTGTCGAGGA<br>GGCCTGCATAATAGACTTTTCCCCTGGGGCAACAAACTGCAGCCCAAAGGCCAGC<br>ATTATGCCAACATTTGGCAGGGCGAGTTTCCGGTGACCAACACTGGTGAGGATGG<br>CTTCCAAGGAACTGCGCCTGTTGATGCCTTCCCTCCCAATGGTTATGGCTTATACA<br>ACATAGTGGGGAACGCATGGGAATGGACTTCAGACTGGTGGACTGTTCATCATTCT<br>GTTGAAGAAACGCTTAACCCAAAAGGTCCCCCTTCTGGGAAAGACCGAGTGAAGA<br>AAGGTGGATCCTACATGTGCCATAGGTCTTATTGTTACAGGTATCGCTGTGCTGCT<br>CGGAGCCAGAACACACCTGATAGCTCTGCTTCGAATCTGGGATTCCGCTGTGCAGC<br>CGACCGCCTGCCCACTATGGACGGCGGAGGGGGCTCCGATTACAAGGACGATGAC<br>GACAAA**TGATAG**ATCGAT
>M6PR	GCGGCCGCC**ATG**TTCCCTTTCTACAGCTGCTGGAGGACTGGACTGCTACTACTACT<br>CCTGGCTGTGGCAGTGAGAGAATCCTGGCAGACAGAAGAAAAAACTTGCGACTTG<br>GTAGGAGAAAAGGGTAAAGAGTCAGAGAAAGAGTTGGCTCTAGTGAAGAGGCTG<br>AAACCACTGTTTAATAAAAGCTTTGAGAGCACTGTGGGCCAGGGTTCAGACACAT<br>ACATCTACATCTTCAGGGTGTGCCGGGAAGCTGGCAACCACACTTCTGGGGCAGG<br>CCTGGTGCAAATCAACAAAAGTAATGGGAAGGAGACAGTGGTAGGGAGACTCAA<br>CGAGACTCACATCTTCAACGGAAGTAATTGGATCATGCTGATCTATAAAGGGGGT<br>GATGAATATGACAACCACTGTGGCAAGGAGCAGCGTCGTGCAGTGGTGATGATCT<br>CCTGCAATCGACACACCCTAGCGGACAATTTTAACCCTGTGTCTGAGGAGCGTGGC<br>AAAGTCCAAGATTGTTTCTACCTCTTTGAGATGGATAGCAGCCTGGCCTGTTCACC<br>AGAGATCTCCCACCTCAGTGTGGGTTCCATCTTACTTGTCACGTTTGCATCACTGGT<br>TGCTGTTTATGTTGTTGGGGGGTTCCTATACCAGCGACTGGTAGTGGGAGCCAAAG<br>GAATGGAGCAGTTTCCCCACTTAGCCTTCTGGCAGGATCTTGGCAACCTGGTAGCA<br>GATGGCTGTGACTTTGTCTGCCGTTCTAAACCTCGAAATGTGCCTGCAGCATATCG<br>TGGTGTGGGGGATGACCAGCTGGGGGAGGAGTCAGAAGAAAGGGATGACCATTT<br>ATTACCAATGAGGAAAAGACGAGGCAGTGGAGAGGGCAGAGGAAGTCTGCTAAC<br>ATGCGGTGACGTCGAGGAGAATCCGGGCCCACTCACCATGCCCGCCATGAAGATC<br>GAGTGCCGCATCACCGGCACCCTGAACGGCGTGGAGTTCGAGCTGGTGGGCGGCG<br>GAGAGGGCACCCCCGAGCAGGGCCGCATGACCAACAAGATGAAGAGCACCAAAG<br>GCGCCCTGACCTTCAGCCCCTACCTGCTGAGCCACGTGATGGGCTACGGCTTCTAC<br>CACTTCGGCACCTACCCCAGCGGCTACGAGAACCCCTTCCTGCACGCCATCAACAA<br>CGGCGGCTACACCAACACCCGCATCGAGAAGTACGAGGACGGCGGCGTGCTGCAC<br>GTGAGCTTCAGCTACCGCTACGAGGCCGGCCGCGTGATCGGCGACTTCAAGGTGG<br>TGGGCACCGGCTTCCCCGAGGACAGCGTGATCTTCACCGACAAGATCATCCGCAG<br>CAACGCCACCGTGGAGCACCTGCACCCCATGGGCGATAACGTGCTGGTGGGCAGC<br>TTCGCCCGCACCTTCAGCCTGCGCGACGGCGGCTACTACAGCTTCGTGGTGGACAG<br>CCACATGCACTTCAAGAGCGCCATCCACCCCAGCATCCTGCAGAACGGGGGCCCC<br>ATGTTCGCCTTCCGCCGCGTGGAGGAGCTGCACAGCAACACCGAGCTGGGCATCG<br>TGGAGTACCAGCACGCCTTCAAGACCCCCATCGCCTTCGCCTAGGGGCCC**TAG**GG<br>GCCC**TAA**ATCGAT

Supplementary Table S2. Results of multiple comparison tests from ARSA expression Western Blotting. Reported p-values were adjusted using the Benjamini–Krieger–Yekutieli (BKY) false discovery rate procedure.

**Two-stage linear step-up procedure of Benjamini, Krieger and Yekutieli**	**Mean diff.**	**Discovery?**	**q value**	**Individual P value**
ARSA:M6PR (1:0) vs. positive control	-0.9337	Yes	<.001	<.001
ARSA:M6PR (1:0) vs. ARSA:M6PR (1:1)	-0.1950	No	.059	.149
ARSA:M6PR (1:0) vs. ARSA:M6PR (4:1)	-0.2818	Yes	.021	.041
ARSA:M6PR (1:0) vs. ARSA:M6PR (8:1)	-0.3719	Yes	.009	.010
ARSA:M6PR (1:0) vs. ARSA:M6PR (16:1)	-0.3593	Yes	.009	.013

Supplementary Table S3. Peak areas for ionisation efficiency testing for modified and unmodified synthetic peptides from ARSA.

Peptide	Retention time (min)	Mass [M+H]+2	Peak Area Rep 1	Peak Area Rep 2	Peak Area Rep 3	Peak Area Rep 4	Peak Area Rep 5	Peak Area Average	Peak Area SD
Unmodified	11.33	866.4273	34314866.3	27986985.5	25881284.0	2.1E+07	31646960.2	2.81E+07	5.32E+06
Iodoacetamide modified	10.8	894.9377	2433611599	2723143660	2981493139	2.6E+09	2600547248	2.66E+09	2.05E+08
fGyl modified	12.23	857.4299	1196002199	1551811263	1436876993	1.8E+09	1276939328	1.46E+09	2.45E+08

Supplementary Table S4. **Relative abundance of peptide species detected in recombinant ARSA samples.** a) Raw area under the curve values from extracted-ion chromatograms b) Raw percent area under the curve for each species. c) Data normalised to account for differences in the ionization efficiencies of the fGly- and IAA-modified peptides.

(a) Raw area under the curve

Peptide sequence	Modification	Control	Rep 1 ARSA: M6PR: FGE			Rep 2 ARSA: M6PR: FGE			Rep 3 ARSA: M6PR: FGE		
			8:1:0	8:1:1	8:1:2	8:1:0	8:1:1	8:1:2	8:1:0	8:1:1	8:1:2
FTDFYVPVSLCTPSR	Unmodified	4.95E+05	2.22E+06	2.51E+07	3.16E+07	1.28E+06	1.04E+07	6.55E+06	1.80E+06	2.72E+07	7.16E+06
FTDFYVPVSLC (+57.02) TPSR	Carbamidomethylation @ Cys69	2.25E+07	2.03E+08	2.78E+07	1.29E+07	1.91E+07	5.70E+07	6.95E+06	7.30E+07	1.01E+07	2.94E+06
FTDFYVPVSLC (-17.99) TPSR	Formylglycine @ Cys69	1.24E+06	5.75E+06	6.54E+07	8.12E+07	4.17E+06	3.24E+07	2.08E+07	6.46E+06	9.12E+07	2.37E+07
FTDFYVPVSLC (+0.0175) TPSR	Hydrated Formylglycine @ Cys69	3.36E+05	1.92E+06	2.41E+07	2.65E+07	1.20E+06	9.65E+06	6.03E+06	1.62E+06	3.23E+07	7.16E+06
Total		2.41E+07	2.11E+08	1.17E+08	1.21E+08	2.44E+07	9.90E+07	3.38E+07	8.10E+07	1.34E+08	3.38E+07

(b) Raw percentage

Peptide sequence	Modification	Control	Rep 1 ARSA: M6PR: FGE			Rep 2 ARSA: M6PR: FGE			Rep 3 ARSA: M6PR: FGE		
			8:1:0	8:1:1	8:1:2	8:1:0	8:1:1	8:1:2	8:1:0	8:1:1	8:1:2
FTDFYVPVSLCTPSR	Unmodified	2.0%	1.0%	17.6%	20.8%	5.0%	9.5%	16.2%	2.2%	16.9%	17.5%
FTDFYVPVSLC (+57.02) TPSR	Carbamidomethylation @ Cys69	91.6%	95.4%	19.5%	8.5%	74.2%	52.1%	17.2%	88.1%	6.3%	7.2%
FTDFYVPVSLC (-17.99) TPSR	Formylglycine @ Cys69	5.0%	2.7%	46.0%	53.3%	16.2%	29.6%	51.6%	7.8%	56.7%	57.8%
FTDFYVPVSLC (+0.0175) TPSR	Hydrated Formylglycine @ Cys69	1.4%	0.9%	16.9%	17.4%	4.7%	8.8%	15.0%	2.0%	20.1%	17.5%

(c) Normalised

Peptide sequence	Modification	Control	Rep 1 ARSA: M6PR: FGE			Rep 2 ARSA: M6PR: FGE			Rep 3 ARSA: M6PR: FGE		
			8:1:0	8:1:1	8:1:2	8:1:0	8:1:1	8:1:2	8:1:0	8:1:1	8:1:2
FTDFYVPVSLCTPSR	Unmodified	1.3%	0.7%	9.1%	10.3%	2.9%	5.2%	8.0%	1.3%	8.2%	8.3%
FTDFYVPVSLC (+57.02) TPSR	Carbamidomethylation @ Cys69	88.9%	93.9%	15.5%	6.5%	67.4%	44.1%	13.1%	83.9%	4.7%	5.3%
FTDFYVPVSLC (-17.99) TPSR	All Formylglycine @ Cys69 species incl hydrated	9.8%	5.4%	75.5%	83.2%	29.7%	50.7%	78.9%	14.8%	87.1%	86.4%

Supplementary Table S5. **Results of multiple comparison tests from Cys-69 modification to Formyl glycine.** Reported p-values were adjusted using the Benjamini–Krieger–Yekutieli (BKY) false discovery rate procedure.

**Two-stage linear step-up procedure of Benjamini, Krieger and Yekutieli**	**Mean diff.**	**Discovery?**	**q value**	**Individual P value**
ARSA:M6PR:FGE (8:1:0) vs. ARSA:M6PR:FGE (8:1:1)	-59.67	Yes	0.001	0.003
ARSA:M6PR:FGE (8:1:0) vs. ARSA:M6PR:FGE (8:1:2)	-74.20	Yes	<0.001	<0.001
ARSA:M6PR:FGE (8:1:1) vs. ARSA:M6PR:FGE (8:1:2)	-14.53	No	0.096	0.275

Supplementary Table S6. **Slopes and R^2 GOF values from end point ARSA enzyme activity assays over a time course of 0 min, 1 min, 5 min, 10 min and 15 min**

Enzyme mass 0.2 µg		
	**Average slope ± std. error**	**GOF (R^2)**
**positive control (+ve)**	0.00006799 ± 0.00003381	0.574
**A:M:F = 8:1:0**	0.00006915 ± 0.00006208	0.9764
**A:M:F = 8:1:1**	0.00168 ± 0.0001609	0.9732
**A:M:F = 8:1:2**	0.002709 ± 0.0002514	0.9748
**negative control (-ve)**	0.000004217 ± 0.000003529	0.3224
**Enzyme mass 0.1 µg**		
	**Average slope ± std. error**	**GOF (R^2)**
**positive control (+ve)**	0.00004278 ± 8.034e-006	0.9043
**A:M:F = 8:1:0**	0.00003675 ± 7.186e-006	0.8971
**A:M:F = 8:1:1**	0.0007481 ± 9.824e-005	0.9508
**A:M:F = 8:1:2**	0.001478 ± 0.0002247	0.9352
**negative control (-ve)**	0.000004233 ± 6.809e-006	0.1142

Supplementary Figures

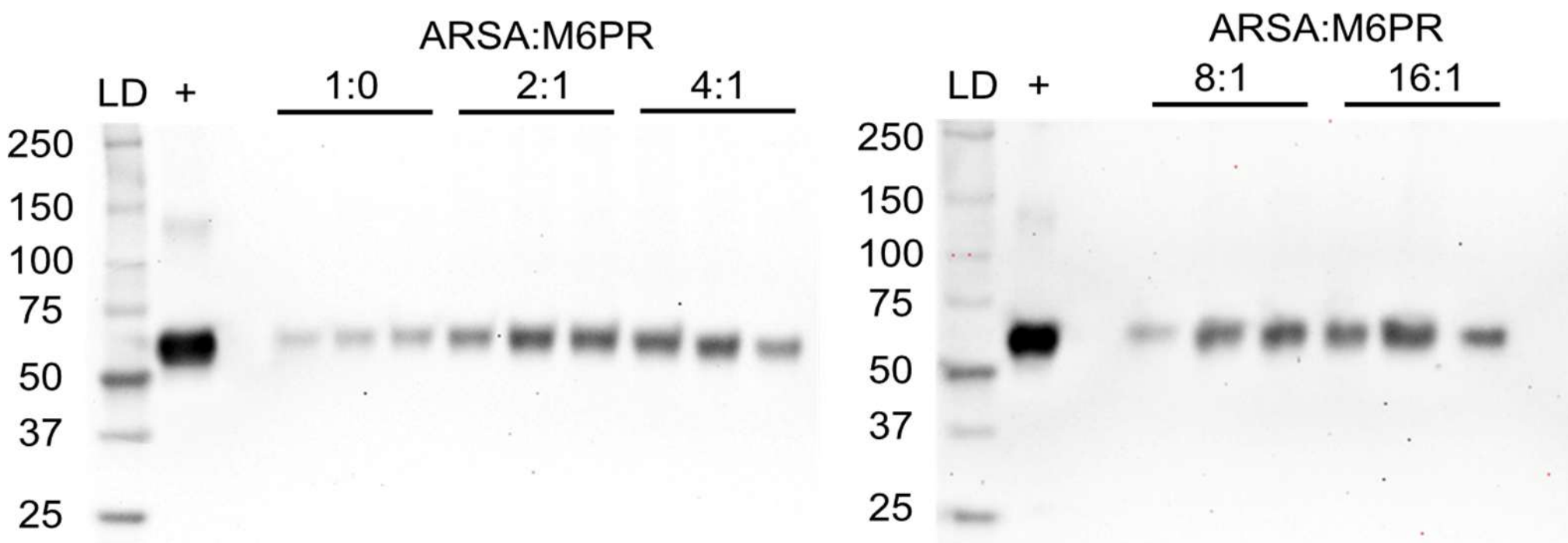

Supplementary Figure S1. Optimisation of M6PR plasmid ratios. Western blot analysis of ARSA co-expression from cell culture supernatant with varied ratios of M6PR for improved cellular export. Strep-Tactin® HRP was used to detect the Twin Strep II tag on ARSA. Each replicate is from an independent transfection (n = 3).

(a) Unmodified peptide

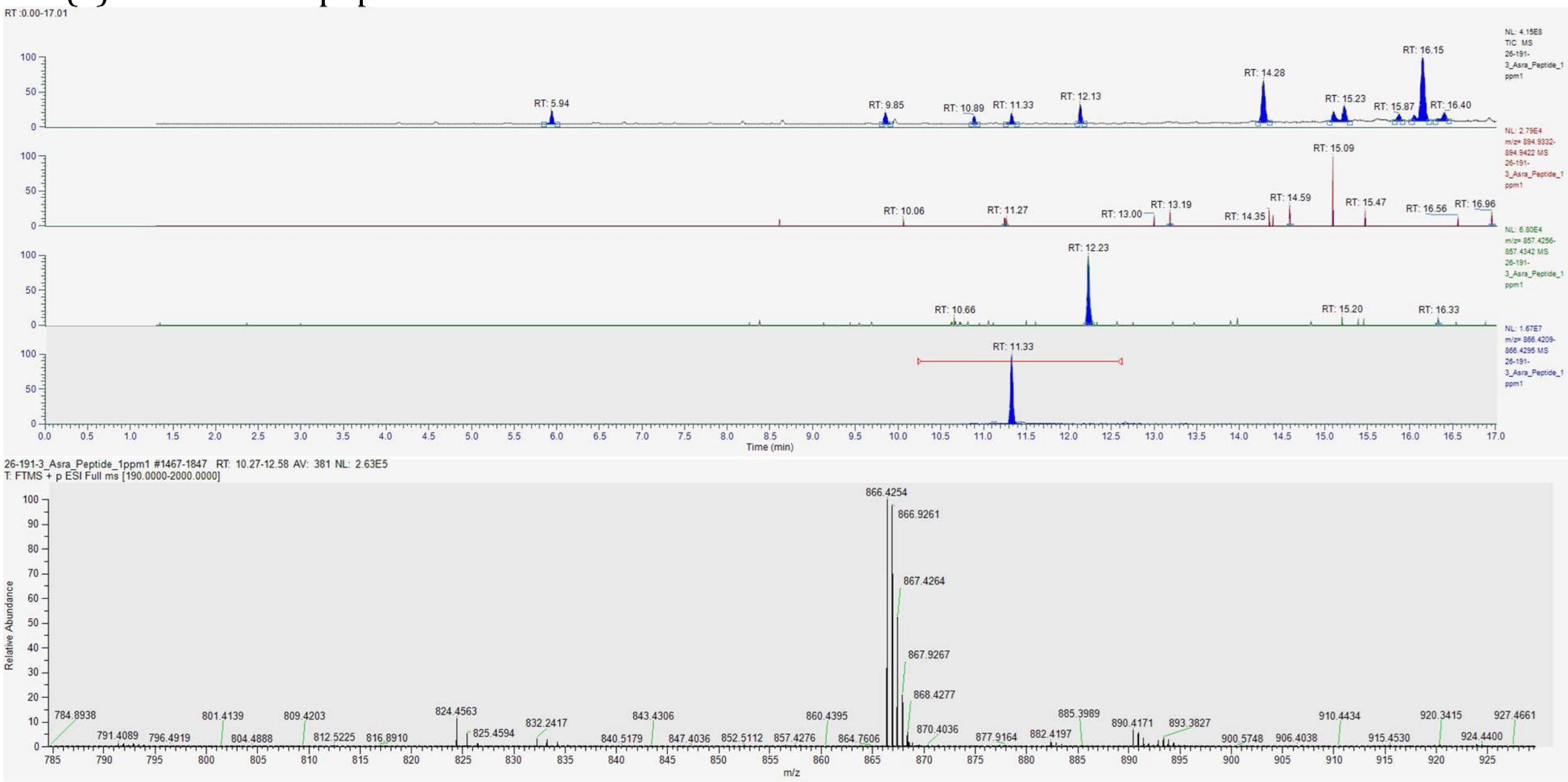

(b) IAA modified

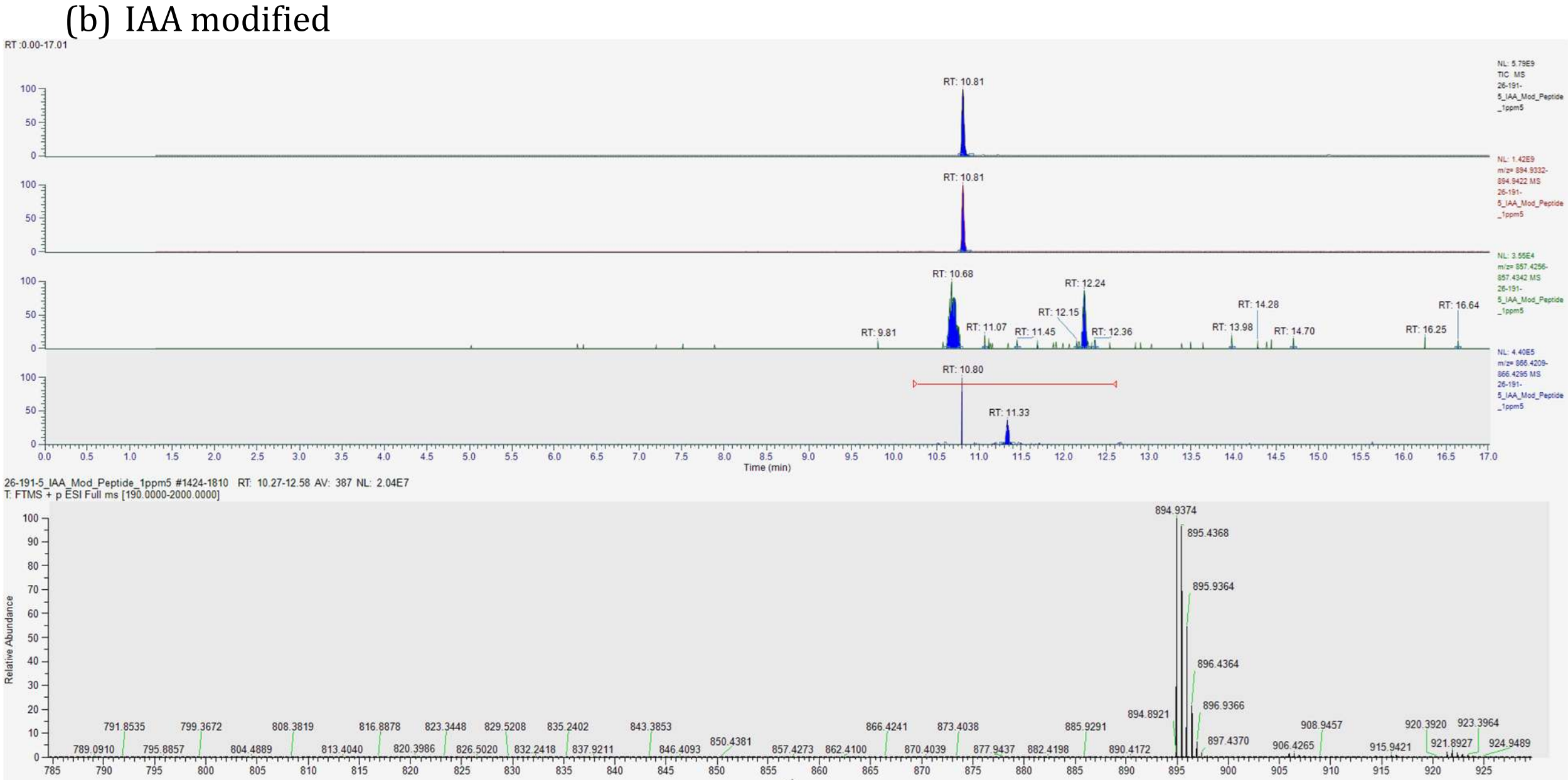

(c) fGly modified

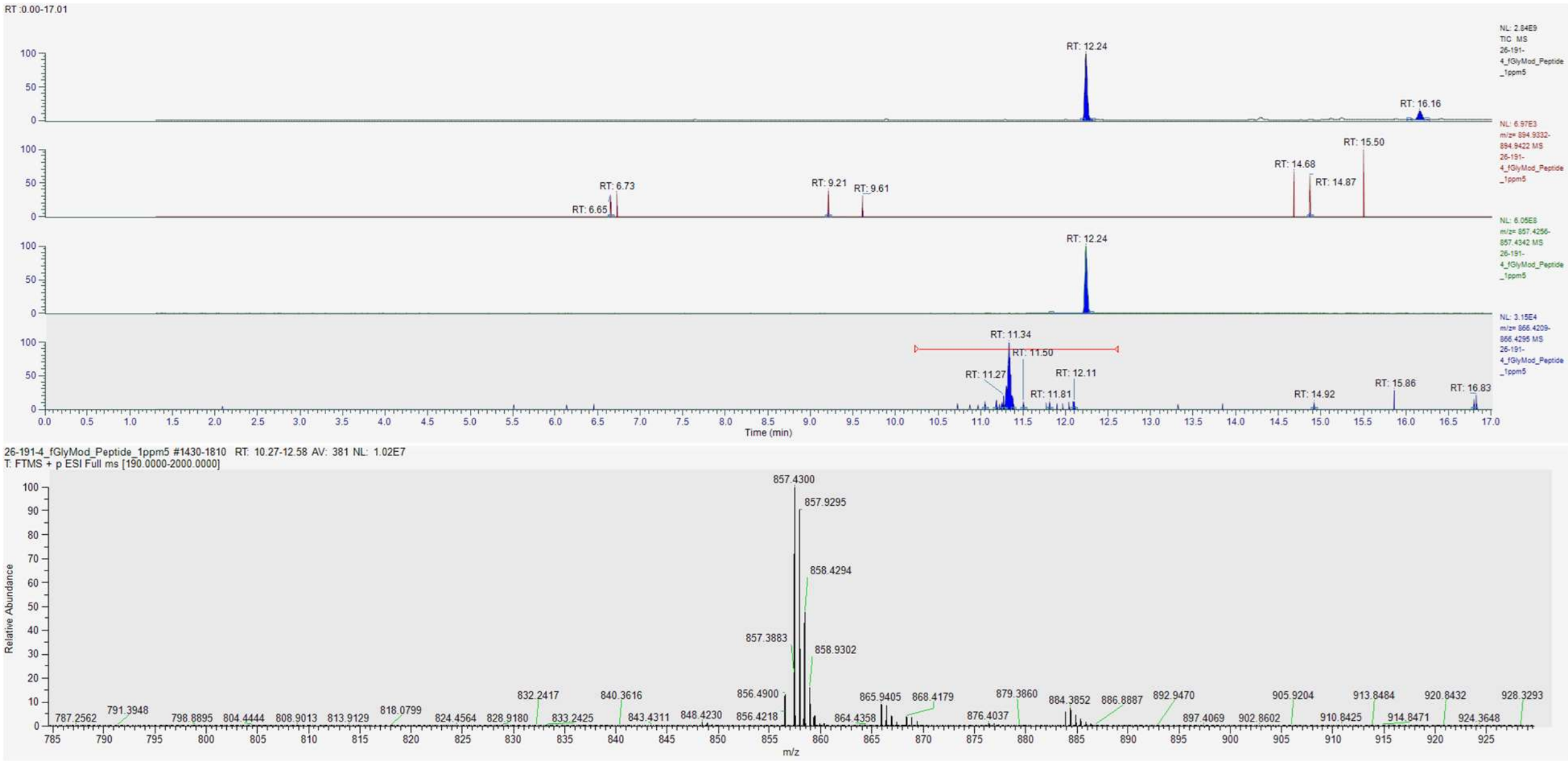

Supplementary Figure S2. Representative extracted ion chromatograms for synthetic peptide controls. Chromatograms are shown for each of the unmodified peptide containing Cys-69 (a), acetylated Cys-69 peptide (b) and fGly modified peptide (c).

(a) 8:1:0

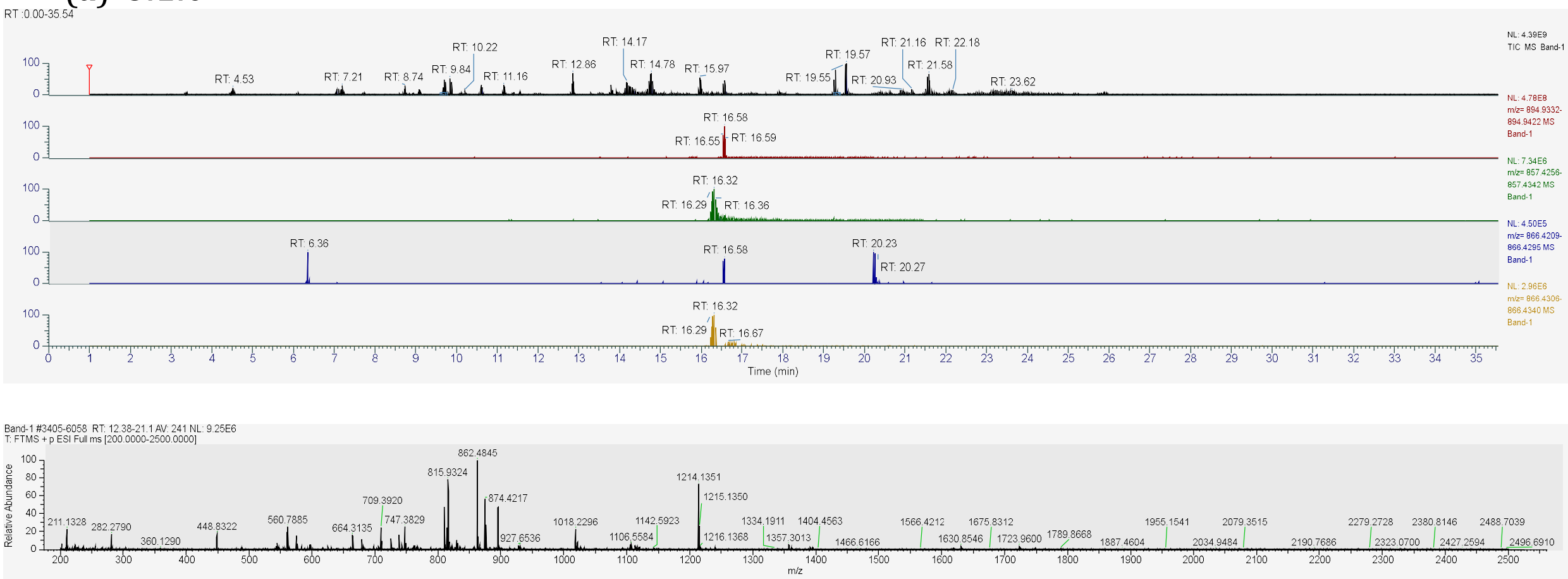

(b) 8:1:1

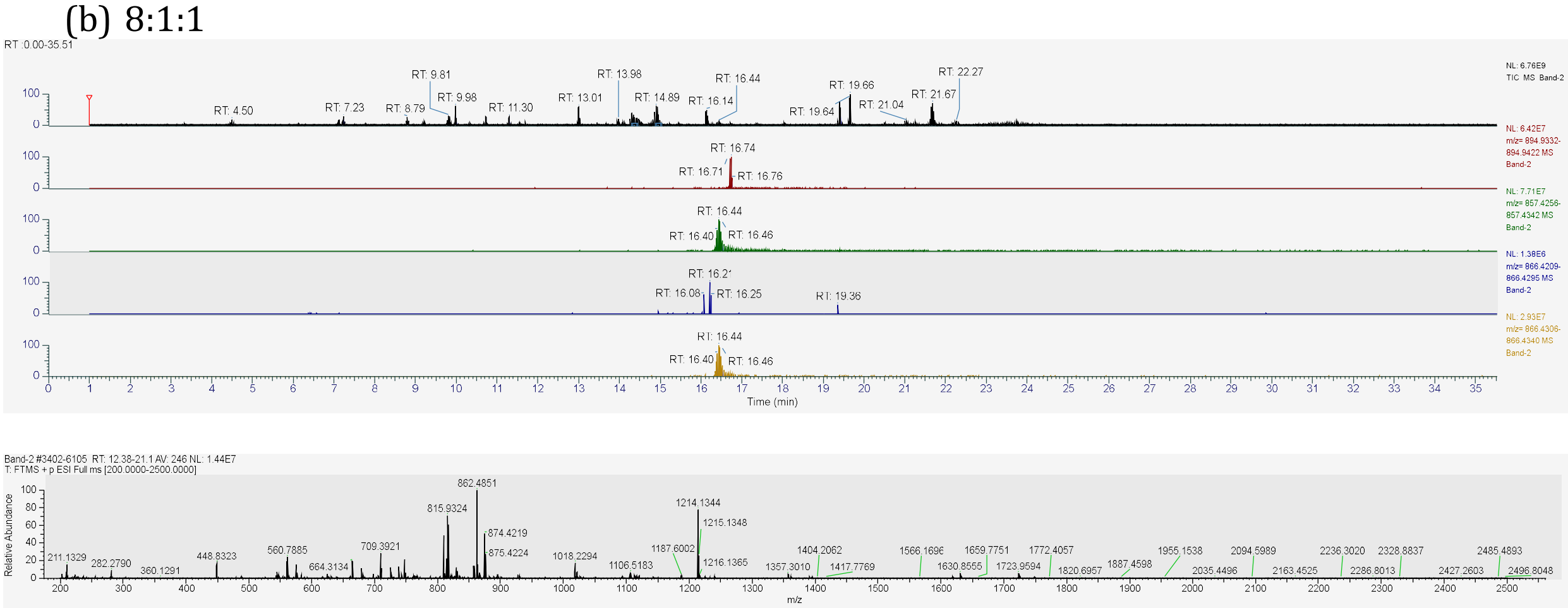

(c) 8:1:2

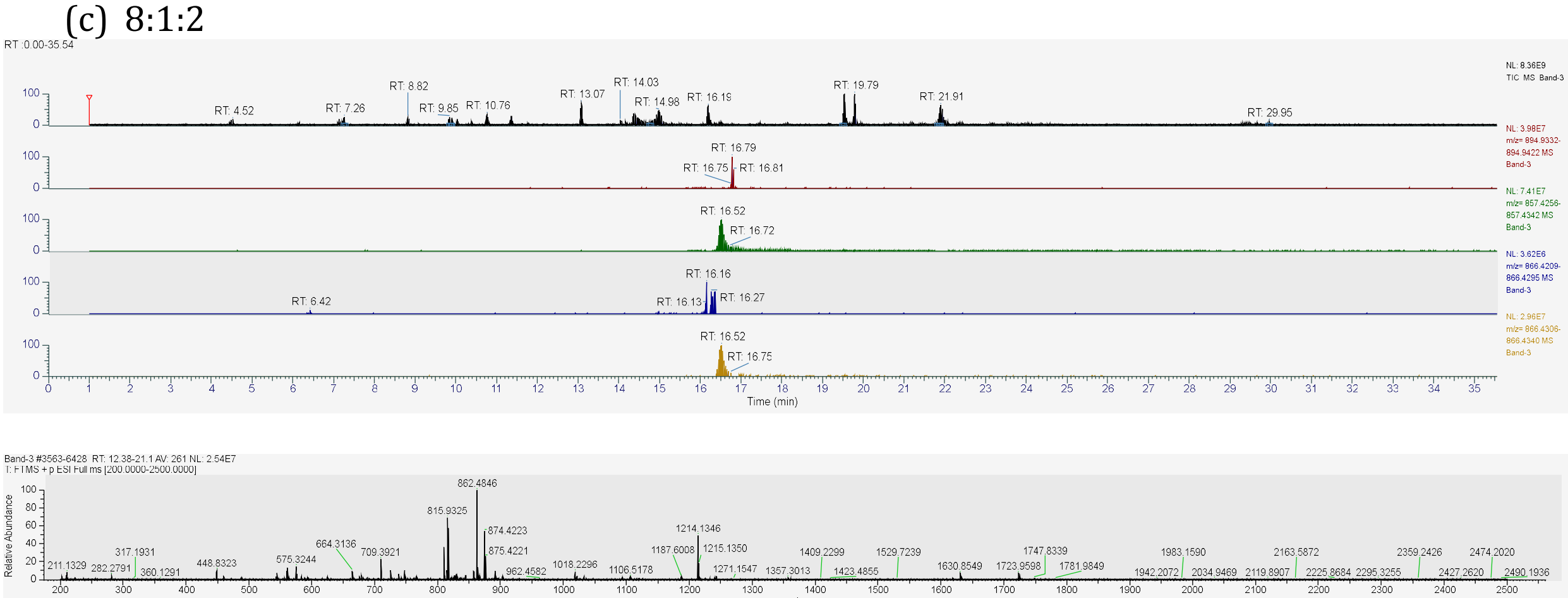

Supplementary Figure S3. Representative extracted ion chromatograms for recombinant ARSA analysis. Chromatograms are shown for a representative replicate for ARSA:M6PR:FGE ratios of (a) 8:1:0, (b) 8:1:1, and (c) 8:1:2.

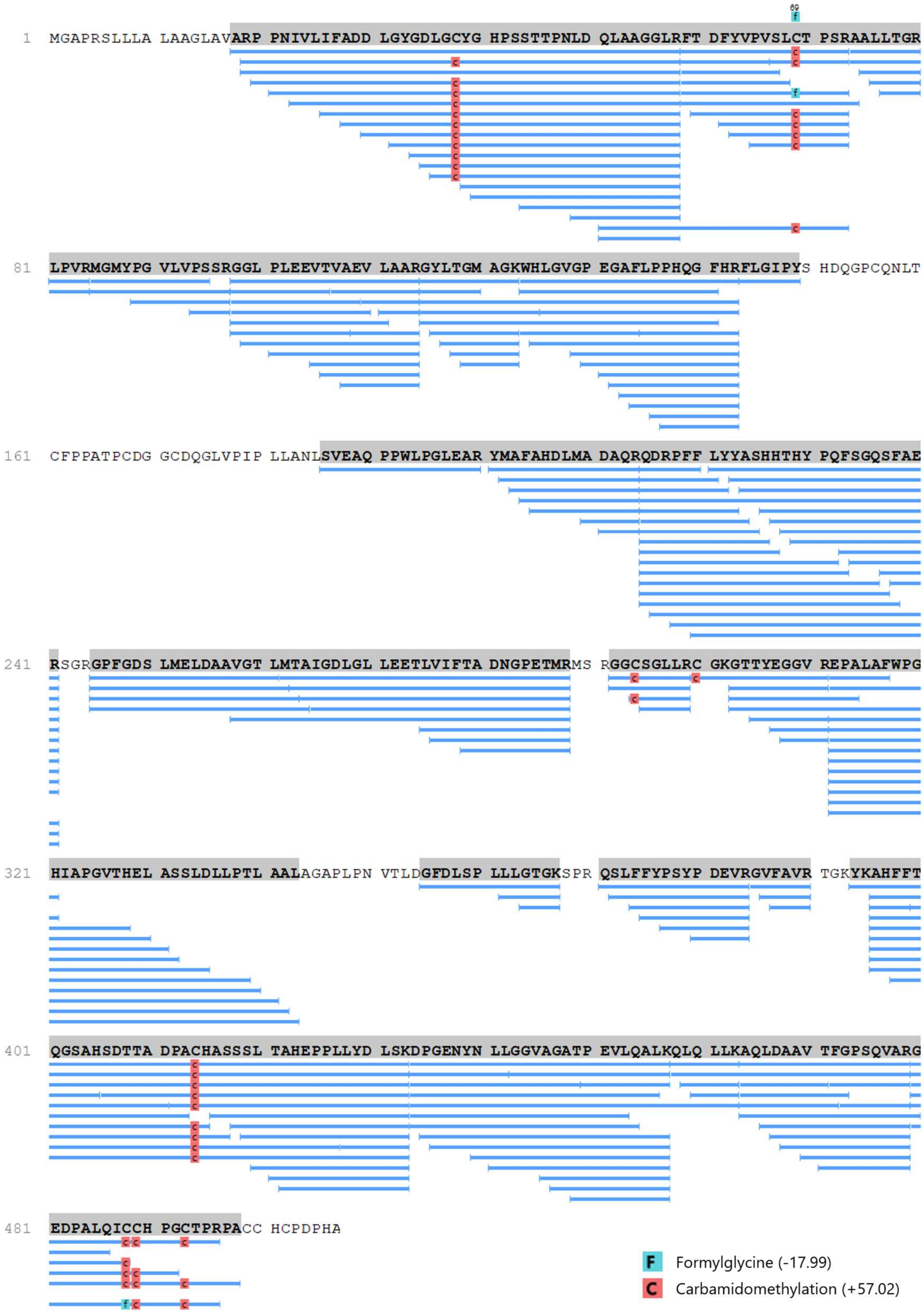

Supplementary Figure S4. Peptide coverage by mass spectrometry of ARSA expressed with an ARSA:M6PR:FGE plasmid ratio of 8:1:0. Cys → fGly modifications are indicated in Green and

Carbamidomethyl modifications are indicated in Red resulting from iodoacetamide treatment of the samples.

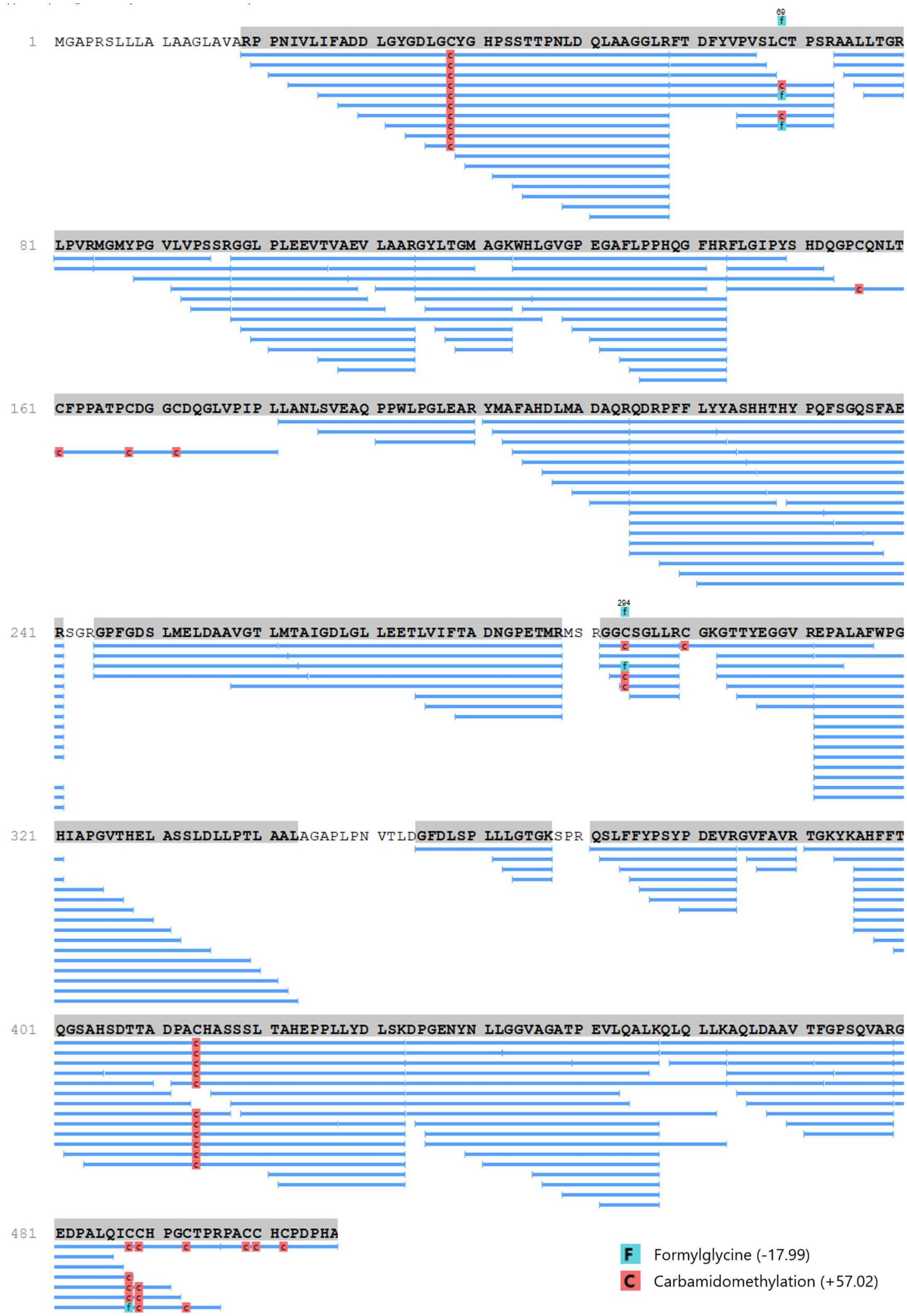

Supplementary Figure S5. Peptide coverage by mass spectrometry of ARSA expressed with an ARSA:M6PR:FGE plasmid ratio of 8:1:1. Cys → fGly modifications are indicated in Green and Carbamidomethyl modifications are indicated in Red resulting from iodoacetamide treatment of the samples.

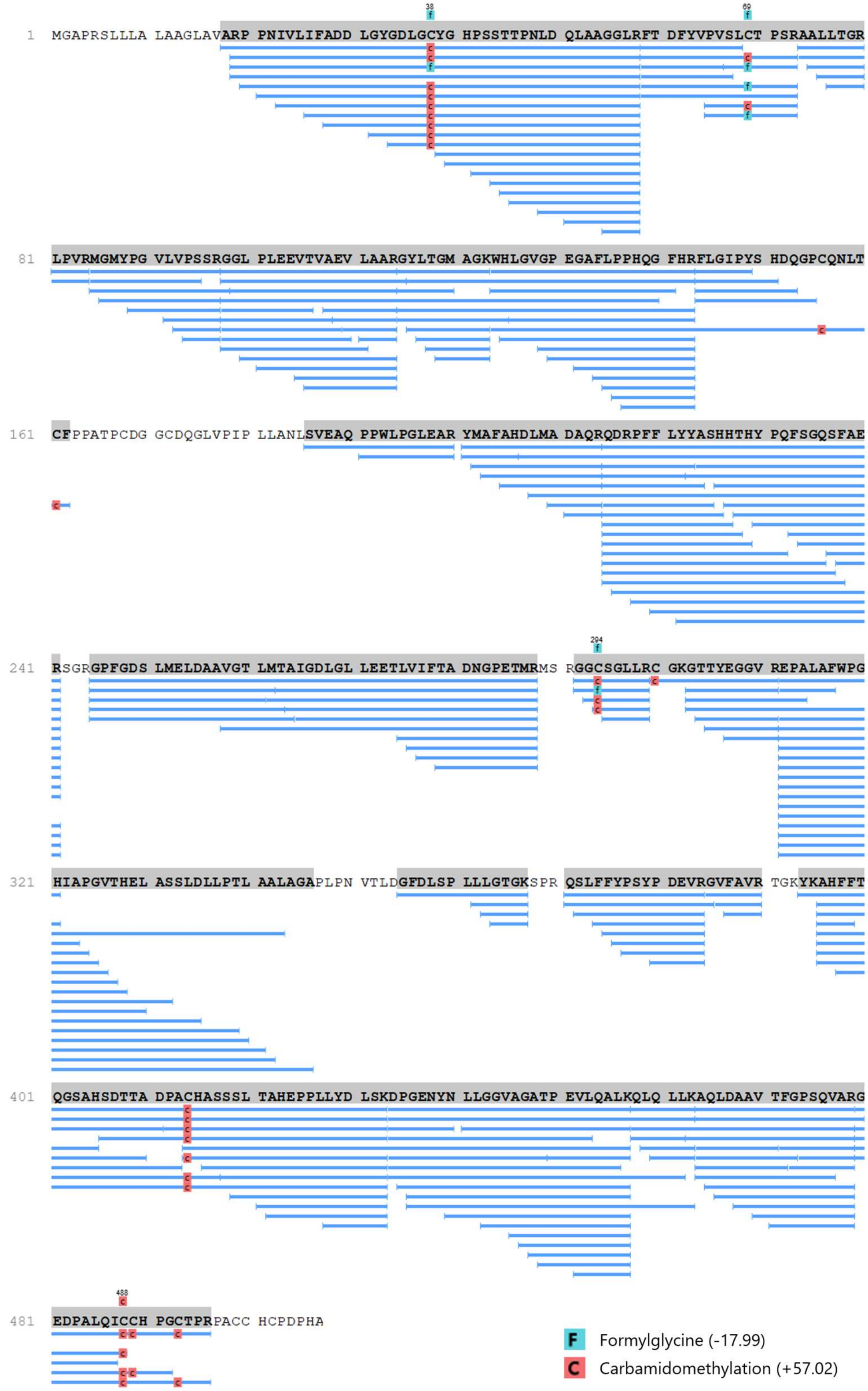

Supplementary Figure S6. Peptide coverage by mass spectrometry of ARSA expressed with an ARSA:M6PR:FGE plasmid ratio of 8:1:2. Cys → fGly modifications are indicated in Green and Carbamidomethyl modifications are indicated in Red resulting from iodoacetamide treatment of the samples.

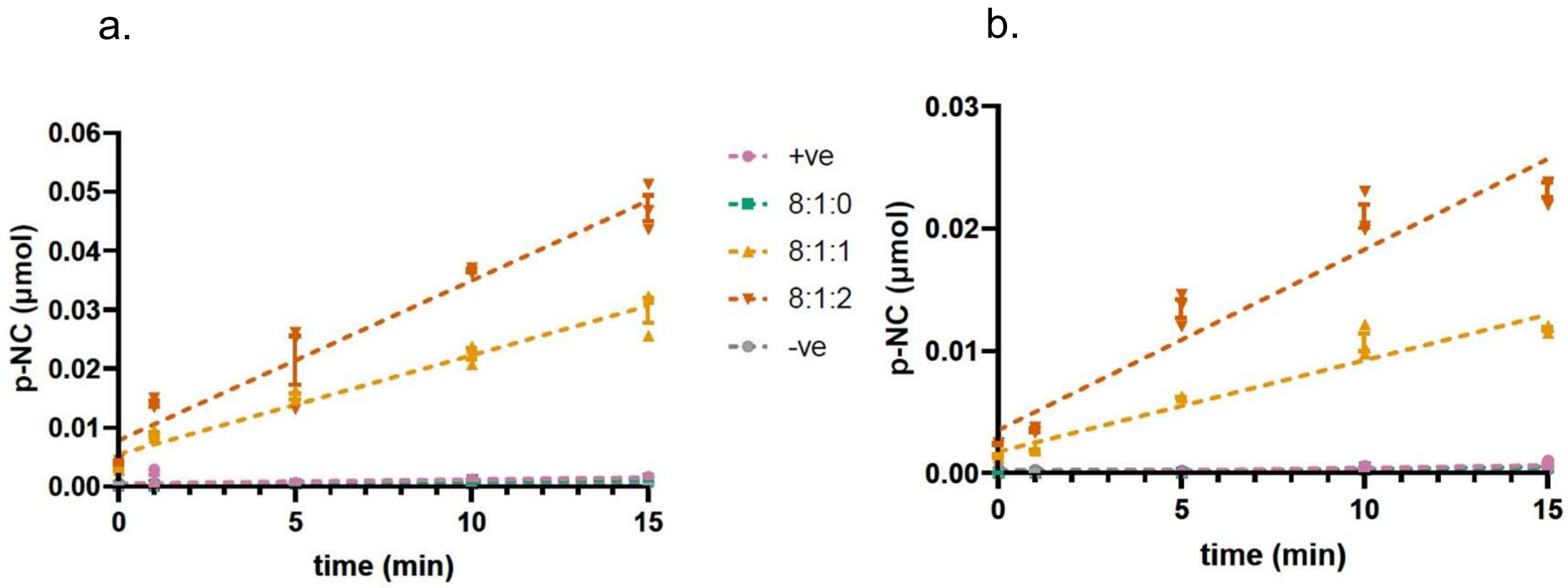

Supplementary Figure S7. **Kinetic characterization of recombinant ARSA.** Time-course activity assays demonstrate the linearity of ARSA-catalyzed reactions at enzyme amounts of (a) 0.2 µg and (b) 0.1 µg. Linear regression was used to obtain slope values for linear regions. Commercially available sulfatase from *Helix pomatia* was included as a positive control for the assay.
